# Survival Benefit and Genetic Profile of Pemetrexed as Initial Chemotherapy in Selected Chinese Patients With Advanced Lung Adenocarcinoma

**DOI:** 10.3389/fonc.2020.01568

**Published:** 2020-09-15

**Authors:** Long-Hua Guo, Ming-Feng Zhang, He-Long Zhang, Jian-Ying Zhou, Xiao-Hong Cai, Yu Long, Qi-Sen Guo, Nong Yang, Jun Zhao, Zhan-Hong Xie, Bo Jiang, Ying Zhu, Yun Fan, Cong-Ying Xie, Yi Hu, Yu Yao, Jun Jia, Xiao-Ling Li, Jiu-Wei Cui, Xi-Zhao Sui, Wen Lin, Ying Cheng, Hui-Juan Wang, Chang-Li Wang, Ming-Fang Zhao, Gui-Bin Qiao, Li-Jun Peng, Lin Yang, Gong-Yan Chen, Kai-Can Cai, Xin-Hua Xu, Liang-Ming Zhang, Guo-Sheng Feng, Jing-Min Zhou, Guo-Wu Wu, Xiao-Rong Dong, Li-Feng Wang, Hong-Mei Zhang, Ya-Jie Gao, Qiu-Ying Jiang, Shun-Dong Cang, Zhi-Xiong Yang, Xia Song, Xiao-Qing Liu, Bo Zhu, Feng-Xia Chen, Chun-Hong Hu, Xi Chen, Yi-Long Wu, Qing Zhou

**Affiliations:** ^1^Department of Medical Oncology, Cancer Center, Meizhou People's Hospital (Huangtang Hospital), Meizhou Academy of Medical Sciences, Meizhou Hospital Affiliated to Sun Yat-Sen University, Meizhou, China; ^2^Guangdong Lung Cancer Institute, Guangdong Provincial Key Laboratory of Translational Medicine in Lung Cancer, Guangdong Provincial People's Hospital, Guangdong Academy of Medical Sciences, School of Medicine, South China University of Technology, Guangzhou, China; ^3^Department of Oncology, The Second Affiliated Hospital of Air Force Military Medical University, Xi'an, China; ^4^Department of Respiratory Disease, Thoracic Disease Center, The First Affiliated Hospital, College of Medicine, Zhejiang University, Hangzhou, China; ^5^Sichuan Cancer Hospital & Institute, Sichuan Cancer Center, School of Medicine, University of Electronic Science and Technology of China, Chengdu, China; ^6^Internal Medicine (Respiratory) of Oncology, Shandong Cancer Hospital Affiliated to Shandong University, Shangdong Academy of Medical Sciences, Shangdong Cancer Hospital and Institute, Jinan, China; ^7^Lung Cancer and Gastrointestinal Unit, Department of Medical Oncology, Hunan Cancer Hospital/Affiliated Cancer Hospital of Xiangya School of Medicine, Changsha, China; ^8^Key Laboratory of Carcinogenesis and Translational Research (Ministry of Education), Department of Thoracic Oncology, Peking University Cancer Hospital and Institute, Beijing, China; ^9^Respiratory Medicine Department, State Key Laboratory of Respiratory Disease, National Clinical Research Center for Respiratory Disease, Guangzhou Institute for Respiratory Health, The First Affiliated Hospital of Guangzhou Medical University, Guangzhou, China; ^10^Department of Cadre Medical Oncology, No.3 Hospital Affiliated to Kunming Medical College (Yunnan Tumor Hospital), Kunming, China; ^11^Department of Thoracic Medical Oncology, Zhejiang Cancer Hospital, Hangzhou, China; ^12^Department of Radiation and Medical Oncology, The First Affiliated Hospital of Wenzhou Medical University, Wenzhou, China; ^13^Department of Medical Oncology, Chinese PLA General Hospital, Beijing, China; ^14^Department of Medical Oncology, The First Affiliated Hospital of Xi'an Jiaotong University, Xi'an, China; ^15^Department of Oncology, Dongguan People's Hospital, Dongguan, China; ^16^Department of Thoracic Oncology, Cancer Hospital of China Medical University, Liaoning Cancer Hospital and Institute, Shenyang, China; ^17^Cancer Center, The First Hospital of Jilin University, Changchun, China; ^18^Department of Thoracic Surgery, Peking University People's Hospital, Beijing, China; ^19^Department of Medical Oncology, Cancer Hospital of Shantou University Medical College, Shantou, China; ^20^Department of Medical Oncology, Jilin Provincial Cancer Hospital, Changchun, China; ^21^Department of Medical Oncology, The Affiliated Cancer Hospital of Zhengzhou University/Henan Cancer Hospital, Zhengzhou, China; ^22^Department of Lung Cancer, Tianjin Medical University Cancer Institute & Hospital, Tianjin, China; ^23^Department of Medical Oncology, The First Hospital of China Medical University, Shenyang, China; ^24^Department of Thoracic Surgery, General Hospital of Southern Theater Command, PLA, Guangzhou, China; ^25^Department of Thoracic Surgery, Shenzhen People's Hospital, 2nd Clinical Medical College of Jinan University, Shenzhen, China; ^26^Department of Medical Oncology, Harbin Medical University Cancer Hospital, Harbin, China; ^27^Department of Thoracic Surgery, Nanfang Hospital of Southern Medical University, Guangzhou, China; ^28^Oncology Department, The First College of Clinical Medical Science, China Three Gorges University & Yichang Central People's Hospital, Yichang, China; ^29^Department of Medical Oncology, Yantai Yuhuangding Hospital, Yantai, China; ^30^Chemotherapy Section One, The People's Hospital of Guangxi Zhuang Autonomous Region, Nanning, China; ^31^Department of Respiratory and Critical Care, Tianjin Chest Hospital, Tianjin, China; ^32^Cancer Center, Union Hospital, Tongji Medical College, Huazhong University of Science and Technology, Wuhan, China; ^33^Oncology Department, Nanjing Drum Tower Hospital, The Affiliated Hospital of Nanjing University Medical School, Nanjing, China; ^34^Department of Clinical Oncology, Xijing Hospital, The Fourth Military Medical University, Xi'an, China; ^35^Department of Oncology, The First Affiliated Hospital of Dalian Medical University, Dalian, China; ^36^Department of Oncology, The Second Hospital Affiliated to Harbin Medical University, Harbin, China; ^37^Department of Oncology, Henan Provincial People's Hospital, Zhengzhou, China; ^38^Department of Pulmonary Oncology, Affiliated Hospital of Guangdong Medical University, Zhanjiang, China; ^39^Department of Respiratory Medicine, Shanxi Provincial Cancer Hospital, Taiyuan, China; ^40^Department of Pulmonary Oncology, The Fifth Medical Centre, Chinese PLA General Hospital, Beijing, China; ^41^Institute of Cancer, Xinqiao Hospital, The Third Military Medical University, Chongqing, China; ^42^Thoracic Surgery, Hainan General Hospital, Haikou, China; ^43^Cancer Center, The Second Xiangya Hospital, Central South University, Changsha, China; ^44^Department of Oncology, The 900th Hospital of the People's Liberation Army Joint Service Support Force, Fuzong Clinical Medical College of Fujian Medical University, Fuzhou, China

**Keywords:** pemetrexed, lung adenocarcinoma, Chinese, next-generation sequencing, chemotherapy

## Abstract

**Objective:** This study investigated survival in selected Chinese patients with advanced lung adenocarcinoma who received initial chemotherapy with pemetrexed. We also explored the relationship between genetic biomarkers and pemetrexed efficacy.

**Methods:** We retrospectively collected patients (*n* = 1,047) enrolled in the Chinese Patient Assistance Program from multiple centers who received pemetrexed alone or combined with platinum as initial chemotherapy and continued pemetrexed maintenance therapy for advanced lung adenocarcinoma from November 2014 to June 2017. The outcomes were duration of treatment (DOT) and overall survival (OS). Clinical features were analyzed for their influence on the treatment effect and prognosis. Next-generation sequencing (NGS) was performed to identify genetic biomarkers associated with the efficacy of pemetrexed.

**Results:** The median DOT was 9.1 months (95% CI: 8.5–9.8), and the median OS was 26.2 months (95% CI: 24.2–28.1). OS was positively correlated with DOT (*r* = 0.403, *P* < 0.001). Multivariable analysis showed that smoking status and Eastern Cooperative Oncology Group (ECOG) performance status (PS) were independently associated with DOT; smoking status, ECOG PS, targeted therapy, and *EGFR/ALK/ROS1* status were independently associated with OS. NGS in 22 patients with available samples showed genes with high mutation rates were: *TP53* (54.5%), *EGFR* (50.0%), *MYC* (18.2%), and *PIK3CA* (13.6%). When grouped based on progression-free survival (PFS) reported in the PARAMOUNT study, the DOT > 6.9 months set was associated with *PIK3CA, ALK, BRINP3, CDKN2A, CSMD3, EPHA3, KRAS*, and *RB1* mutations, while *ERBB2* mutation was observed only in the DOT ≤ 6.9 months set.

**Conclusion:** This study shows that initial chemotherapy with pemetrexed is an effective regimen for advanced lung adenocarcinoma in selected Chinese patients. There is no specific genetic profile predicting the benefit of pemetrexed found by NGS. Biomarkers predicting the efficacy of pemetrexed need further exploration.

## Introduction

Lung cancer is the most commonly diagnosed cancer and the leading cause of cancer death all over the world (1). About 80–85% of human lung cancers belong to the category of non-small cell lung cancer (NSCLC). These patients usually present with locally advanced (stage IIIB) or metastatic disease (stage IV) (2). Immunotherapy plus chemotherapy have become a standard first-line treatment for patients with no oncogenic driver alterations in advanced NSCLC. Nevertheless, which chemotherapy regimen combined with immunotherapy agents, will achieve optimal outcomes for these patients is unknown. In addition, in developing countries, including China, immunotherapy is often too expensive for many patients, although these drugs are recommended by the available guidelines (3). Therefore, chemotherapy is still indispensable (4–6). Some randomized controlled trials and real-world studies abroad demonstrated that pemetrexed-cisplatin induction and pemetrexed maintenance therapy is an effective and well-tolerated regimen in stage IIIB-IV patients with non-squamous NSCLC (7–14), but there is still a lack of large sample size evidence to report the survival benefit of pemetrexed as the initial chemotherapy in Chinese patients with advanced lung adenocarcinoma.

Moreover, heterogeneity in response to pemetrexed has been observed, some patients experience long progression-free survival (PFS) or overall survival (OS), whereas others are having short PFS and OS (15). Identifying patients who are unlikely to benefit from pemetrexed would avoid unnecessary treatment and allow alternative therapy that might achieve better outcomes. Although we know that histologically non-squamous NSCLC is a predictive factor for the efficacy of pemetrexed, this still cannot help us screen out the patients who will really benefit from pemetrexed (16). Previous studies showed that thymidylate synthase (TS), folate receptor alpha (FRA), epidermal growth factor receptor (*EGFR*) gene mutation, anaplastic lymphoma kinase (*ALK*) gene rearrangement, and plasma microRNA levels might be predictive markers for pemetrexed (17–22), but the predictability of these biomarkers has not been reproducible due to the lack of prospective studies. Therefore, it is necessary to explore novel and reliable biomarkers to predict the effect of pemetrexed.

Next-generation sequencing (NGS) based on tumor tissue or liquid biopsy has begun to play a role in genomic profiling. Its high-throughput nature makes testing of thousands of genes or even the whole genomes possible with a small amount of DNA, allowing this method to identify actionable genomic alterations (23).

In order to reduce the financial burden and provide timely treatment for Chinese patients, the Chinese Primary Health Care Foundation launched a patient assistance program (PAP) for advanced non-squamous NSCLC patients with pemetrexed as maintenance therapy; those patients receive a 100% discount after a self-funded four-cycle induction pemetrexed therapy, starting from October 1, 2014. The patients who do not complete the four-cycle induction pemetrexed therapy are not eligible for the PAP. Following the implementation of the PAP, we observed the duration of treatment (DOT) with pemetrexed and OS among these selected patients and compared the genomic differences between the patients with long and short duration of pemetrexed treatment to explore potential predictive biomarkers.

## Methods

### Study Design

This was a retrospective study of data from multiple centers across China. The patients were funded by the PAP to receive pemetrexed and visited their treating hospitals from November 2014 to June 2017. The PAP is offered by the Chinese Primary Health Care Foundation. All data were extracted from the patients' medical records, and telephone follow-up was conducted by physicians across more than 200 tertiary hospitals in China. The study protocol was approved by the Research Ethics Committee of Guangdong Provincial People's Hospital, Guangdong Academy of Medical Sciences, School of Medicine, South China University of Technology (No. GDREC2017303H). This study was conducted in accordance with the Good Clinical Practice (GCP) principles. Written informed consent was obtained from all included patients.

### Study Population

The patients meeting the following criteria were included: Eastern Cooperative Oncology Group (ECOG) performance status (PS) of 0–2; lung adenocarcinoma; stage IV (according to the American Joint Committee on Cancer staging system, 7th edition); pemetrexed as initial chemotherapy; received four cycles of pemetrexed monotherapy or pemetrexed plus platinum as induction chemotherapy with no disease progression according to Response Evaluation Criteria in Solid Tumors (RECIST), version 1.1 (24): complete response (CR), partial response (PR), stable disease (SD), or progressive disease (PD); and at least one cycle of pemetrexed maintenance therapy from PAP.

The exclusion criteria were: history of chemotherapy; combination with other antitumor drugs such as bevacizumab; disease progression before the completion of four cycles of induction pemetrexed chemotherapy; or unavailable treatment information or survival data.

### Data Source

The demographic and clinical characteristics of the patients were extracted from their medical records and entered into the Medical Record Abstraction Form (MERAF) by designated hospital staff. These characteristics included sex, age, smoking status, disease stage at diagnosis, the time at diagnosis, disease stage at pemetrexed treatment, NSCLC histological type, gene status, ECOG PS, the time of pemetrexed treatment initiation, first-line anticancer treatment regimen, cycles, the best response to pemetrexed treatment, the time of progressive disease administered with pemetrexed, other treatment after pemetrexed, survival status, and the time to death. Patients who had smoked ≥100 cigarettes in their lifetime were defined as smokers (25). The last time of pemetrexed treatment was obtained through the electronic PAP system. Survival data were collected by the follow-up registration system from each site and by telephone follow-up. The response was assessed based on imaging examination reports and medical case notes.

### Outcomes

The outcomes were the DOT of pemetrexed and OS. DOT was defined as the time from the initiation to the last pemetrexed chemotherapy. OS was defined as the time from the initiation of pemetrexed chemotherapy to death or the last follow-up, whichever came first. The last follow-up was conducted on March 31, 2018.

### Grouping

The median PFS was 6.9 months for patients with advanced non-squamous NSCLC who received maintenance therapy with pemetrexed after induction chemotherapy with pemetrexed plus cisplatin according to the double-blind, phase 3, randomized controlled trial “PARAMOUNT” (9). Since the PARAMOUNT study provided high-level evidence for maintenance therapy with pemetrexed, we selected their median PFS as the cutoff for grouping. In the present study, according to their PFS, patients with NGS detection whose DOT was ≤ 6.9 months were assigned to the short duration group, whereas the long duration group included patients with DOT of >6.9 months.

### Next-Generation Sequencing

Patients with available blood samples at Guangdong Provincial People's Hospital underwent NGS. Plasma samples were obtained after disease progression in patients with initial pemetrexed chemotherapy. The NGS tests targeted at least 139 genes related to lung cancer and were performed in two clinical testing centers (Burning Rock Biotech Ltd and Nanjing Geneseeq Technology Inc.). First, DNA was extracted from blood. Then, the NGS library was prepared, and DNA was profiled using a capture-based sequencing panel. Finally, sequence data were analyzed and compared between the long and short duration groups.

### Statistical Analysis

SPSS 22.0 (IBM Corp., USA) was used for all statistical analyses. Descriptive statistics were used to describe the enrolled patients. DOT and OS were assessed using the Kaplan-Meier method. We also performed the Pearson correlation test to evaluate the correlation between DOT and OS. The factors associated with DOT and OS were analyzed by performing univariable and multivariable analyses using Cox proportional hazards models, including the following covariables: age, sex, smoking status, ECOG PS, and gene status. Multivariable analysis of OS also included the factor: targeted therapy. Variables with *P* < 0.1 in the univariable analyses were included in the multivariable analysis. Two-sided *P* < 0.05 was considered statistically significant.

## Results

### Demographics and Clinical Characteristics

**Figure 1:**
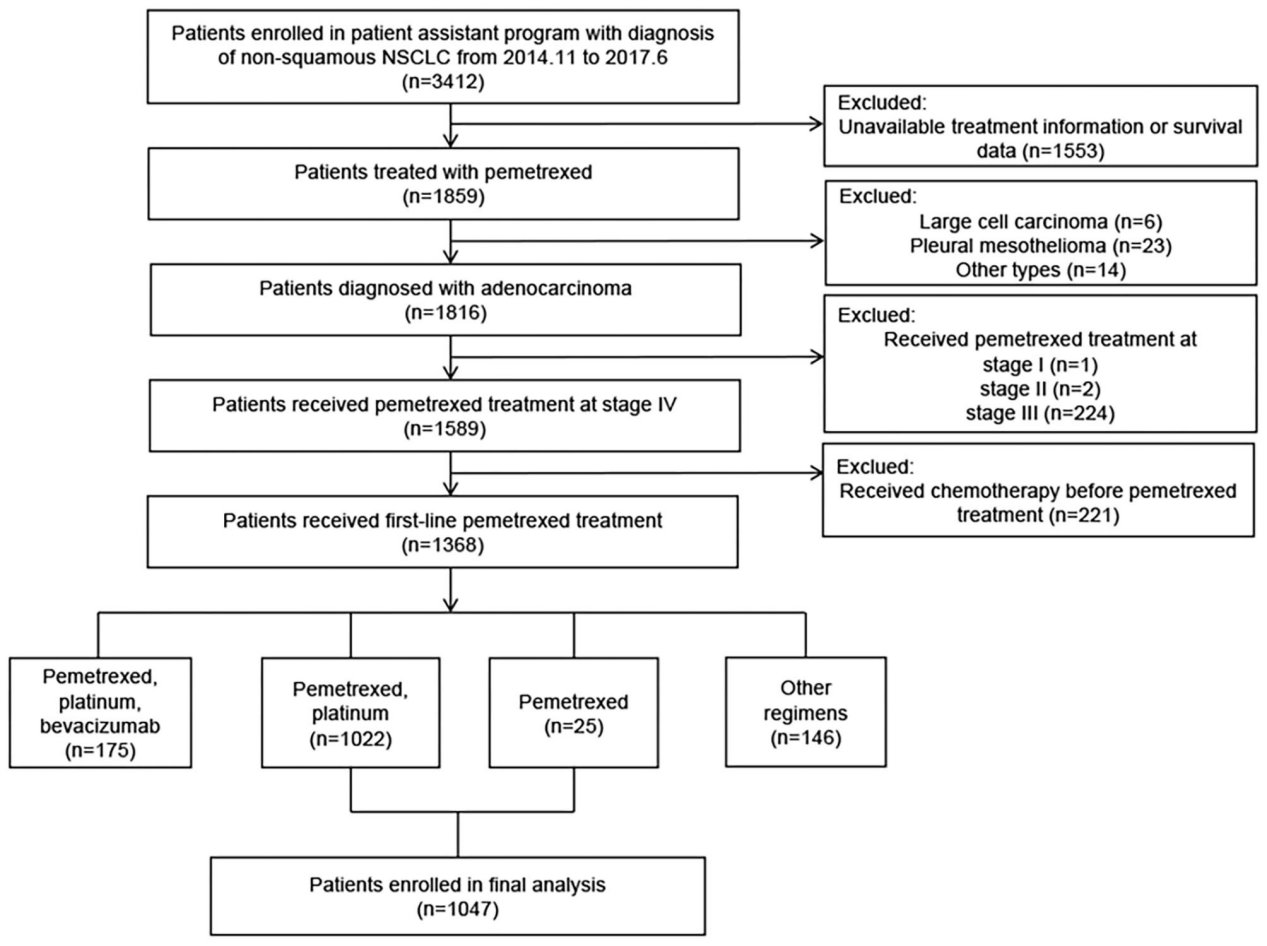
Flowchart of patients' screening for inclusion in the study.

A total of 3,412 patients were screened. Of these, 1,047 patients from 44 hospitals with advanced lung adenocarcinoma who received pemetrexed treatment were included in the analyses. The screening flowchart and clinical characteristics of the patients are presented in Figure 1, Table 1, respectively.

**Table 1:** Demographic and clinical characteristics of patients with advanced lung adenocarcinoma.

**Characteristics**	**Patients (*n* = 1,047)**
**Sex**, ***n*** **(%)**	
Male	594 (56.7%)
Female	453 (43.3%)
**Age (years)**	
Median (range)	59 (24–93)
<65, *n* (%)	758 (72.4%)
≥65, *n* (%)	289 (27.6%)
**Smoking status**, ***n*** **(%)**	
Non-smoker	608 (58.1%)
Smoker	439 (41.9%)
**ECOG PS**, ***n*** **(%)**	
0–1	984 (94.0%)
2	63 (6.0%)
**Targeted therapy before pemetrexed treatment**, ***n*** **(%)**	
Yes	149 (14.2%)
No	898 (84.8%)
**Gene status before pemetrexed treatment**, ***n*** **(%)**	
***EGFR*** **Mutation**	
Positive	266 (25.4%)
Negative	568 (54.3%)
Unknown	213 (20.3%)
***ALK*** **Rearrangement**	
Positive	59 (5.6%)
Negative	580 (55.4%)
Unknown	408 (39.0%)
***KRAS*** **Mutation**	
Positive	11 (1.1%)
Negative	147 (14.0%)
Unknown	889 (84.9%)
***ROS1*** **Fusion**	
Positive	18 (1.7%)
Negative	233 (22.3%)
Unknown	796 (76.0%)
**Chemotherapy regimen**, ***n*** **(%)**	
Pemetrexed plus platinum	1,022 (97.6%)
Pemetrexed monotherapy	25 (2.4%)
**Best tumor response**, ***n*** **(%)**	
PR	477 (45.6%)
SD	570 (54.4%)

*ECOG PS, Eastern Cooperative Oncology Group performance status; PR, partial response; SD, stable disease*.

The gene status before pemetrexed treatment was analyzed, including *EGFR, ALK*, Kirsten rat sarcoma viral oncogene homolog (*KRAS*), and c-ros oncogene 1 (*ROS1*). The gene aberration rates in patients with definitive results were 31.9% (266/834) for *EGFR* mutation, 9.2% (59/639) for *ALK* rearrangement, 7.0% (11/158) for *KRAS* mutation, and 7.2% (18/251) for *ROS1* fusion (Table 2).

**Table 2:** The gene aberration rates in patients with definitive results.

**Gene aberration**	**Positive, *n***	**Negative, *n***	**Mutation rate, %**
*EGFR*	266	568	31.9%
*ALK*	59	580	9.2%
*ROS1*	18	233	7.2%
*KRAS*	11	147	7.0%

### Survival Outcomes

**Figure 2:**
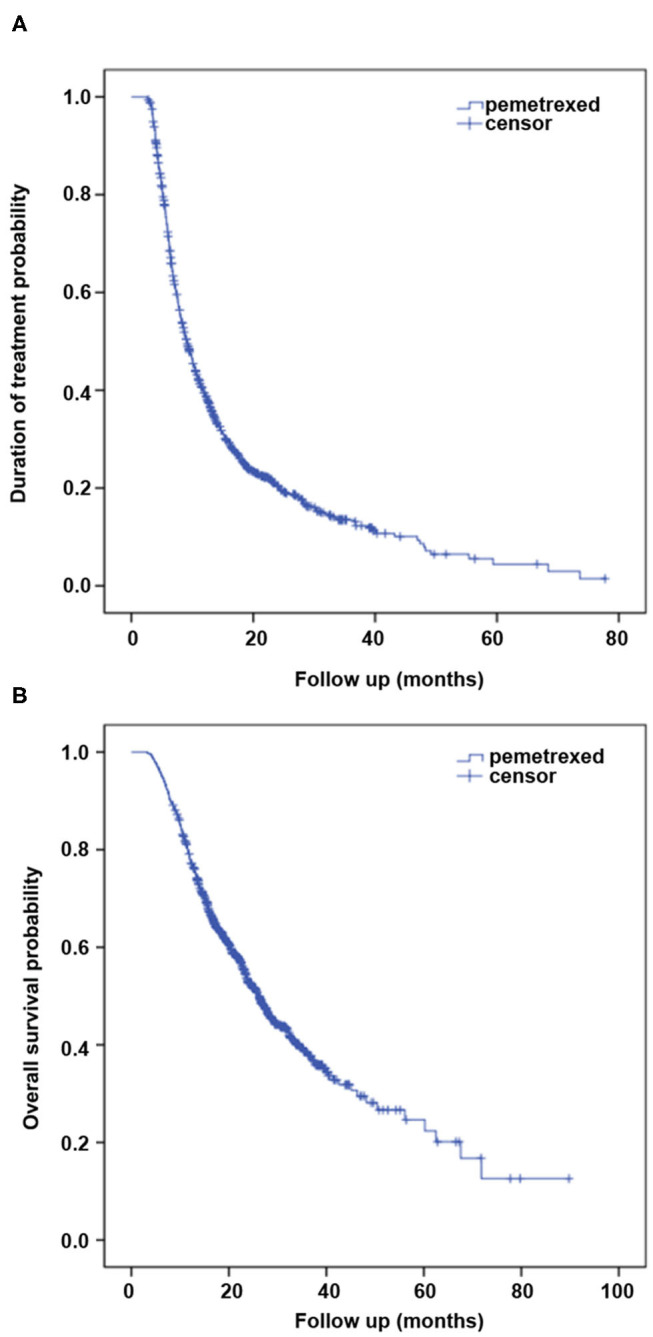
Kaplan–Meier curves of **(A)** duration of treatment (DOT) and **(B)** overall survival (OS) for 1,047 patients treated with pemetrexed.

The median follow-up of all patients was 19.1 months. The median DOT was 9.1 months (95% confidence interval [CI]: 8.5–9.8) for 811 patients who had stopped pemetrexed treatment at the last follow-up (Figure 2A). Among the 536 patients who had died, the median OS was 26.2 months (95%CI: 24.2–28.1) (Figure 2B). Moreover, a positive correlation was observed between DOT defined in the present study and OS evaluated by Pearson correlation test (*r* = 0.543, *P* < 0.001).

DOT and OS were both longer than the PFS and OS observed in the PARAMOUNT, JMII, and S110 trials. Clinical characteristics and efficacy of pemetrexed in the present study were compared with these trials (Supplemental Table 1).

### Factors Associated With Duration of Treatment and Overall Survival

**Figure 3:**
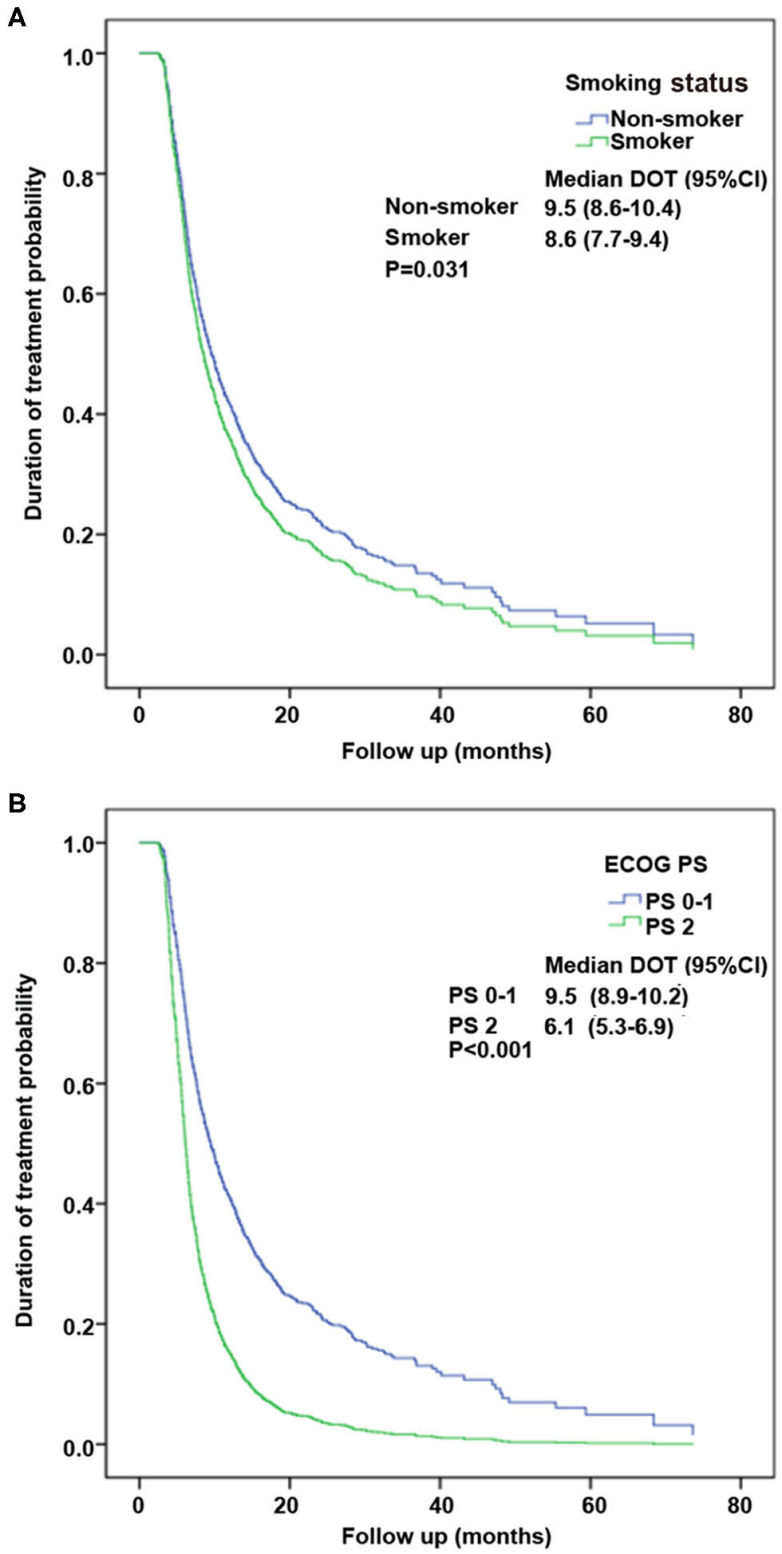
Kaplan–Meier curves of the duration of treatment (DOT) about the significant factors by multivariate analysis. **(A)** Performance status (PS) factor. **(B)** Smoking status factor.

When the variables were analyzed by univariable analysis, sex, smoking status, ECOG PS, and gene status (*ALK, KRAS, ROS1*) were revealed as significant factors (*P* < 0.1) associated with DOT. These parameters were included in the multivariable Cox regression analysis. As a result, only smoking status (hazard ratio [HR], 1.167; 95%CI, 1.015–1.342; *P* = 0.031) and ECOG PS (HR, 2.112; 95% CI, 1.631–2.735; *P* < 0.001) were independent factors influencing DOT of pemetrexed (Table 3). Kaplan–Meier curves of DOT about smoking status and ECOG PS factors are shown in Figure 3.

**Table 3:** Univariable and multivariable analysis of duration of pemetrexed treatment.

**Variable**	**Univariable analysis**				**Multivariable analysis**			
	**HR**	**95% CI**		***P***	**HR**	**95% CI**		***P***
		**Lower**	**Upper**			**Lower**	**Upper**	
Age (≥65 vs. <65 years)	1.045	0.896	1.219	0.574	-	-	-	-
Sex (female vs. male)	0.889	0.772	1.022	0.099	-	-	-	0.642
Smoking status (smoker vs. non-smoker)	1.163	1.011	1.338	0.034	1.167	1.015	1.342	0.031
ECOG PS (2 vs. 0–1)	2.105	1.626	2.726	<0.001	2.112	1.631	2.735	<0.001
***EGFR*** **Mutation**								
(+ vs. -)	1.071	0.909	1.261	0.413	-	-	-	-
(unknown vs. -)	0.976	0.814	1.171	0.795	-	-	-	-
***ALK*** **Rearrangement**								
(+ vs. -)	0.716	0.520	0.987	0.041	-	-	-	0.056
(unknown vs. -)	1.000	0.866	1.155	0.996	-	-	-	0.943
***KRAS*** **Mutation**								
(+ vs. -)	1.754	0.919	3.348	0.088	-	-	-	0.117
(unknown vs. -)	1.069	0.878	1.301	0.509	-	-	-	0.788
***ROS1*** **Fusion**								
(+ vs. -)	0.604	0.343	1.063	0.080	-	-	-	0.096
(unknown vs. -)	1.010	0.855	1.192	0.911	-	-	-	0.762

*HR, hazard ratio; CI, confidence interval; ECOG PS, Eastern Cooperative Oncology Group performance status*.

**Figure 4:**
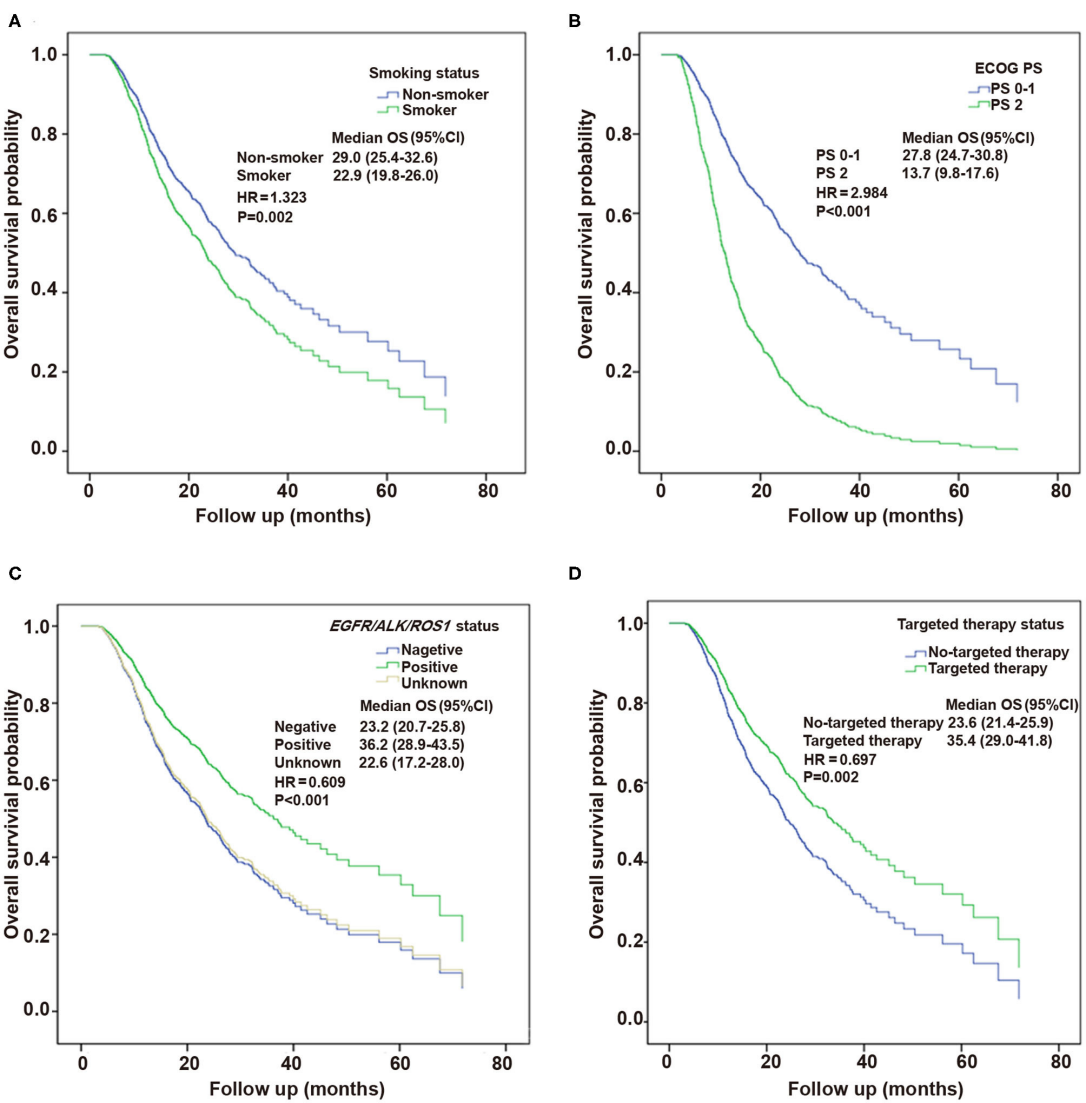
Kaplan–Meier curves of overall survival (OS) about the significant factors by multivariate analysis. **(A)** Smoking status factor. **(B)** Performance status (PS) factor. **(C)** *EGFR/ALK/ROS1* mutation factor. **(D)** Targeted therapy factor.

Multivariable analysis in the Cox proportional hazards model revealed the independent factors influencing OS, including smoking status (HR, 1.323; 95% CI, 1.111–1.574; *P* = 0.002), ECOG PS (HR, 2.984; 95% CI, 2.286–3.894; *P* < 0.001), targeted therapy (HR, 0.697; 95% CI, 0.556–0.875; *P* = 0.002), and *EGFR/ALK/ROS1* status (HR, 0.609; 95% CI, 0.552–0.863; *P* < 0.001) (Table 4). Figure 4 presents the Kaplan–Meier curves of OS about these significant factors: smoking status, ECOG PS, *EGFR/ALK/ROS1*, and targeted therapy factors.

**Table 4:** Univariable and multivariable analysis of overall survival.

**Variable**	**Univariable analysis**				**Multivariable analysis**			
	**HR**	**95% CI**		***P***	**HR**	**95% CI**		***P***
		**Lower**	**Upper**			**Lower**	**Upper**	
Age (≥65 vs. <65 years)	1.242	1.031	1.495	0.022	-	-	-	0.093
Sex (female vs. male)	0.731	0.614	0.870	<0.001	-	-	-	0.267
Smoking status (smoker vs. non-smoker)	1.416	1.194	1.680	<0.001	1.323	1.111	1.574	0.002
ECOG PS (2 vs. 0–1)	2.934	2.254	3.821	<0.001	2.984	2.286	3.894	<0.001
***EGFR/ALK/ROS1*** **Status**								
(+ vs. -)	0.569	0.463	0.699	<0.001	0.609	0.552	0.863	<0.001
(unknown vs. -)	1.023	0.822	1.274	0.838	0.979	0.785	1.221	0.852
Targeted therapy (with vs. without)	0.599	0.485	0.740	<0.001	0.697	0.556	0.875	0.002

*HR, hazard ratio; CI, confidence interval; ECOG PS, Eastern Cooperative Oncology Group performance status*.

### Genetic Differences Between the Long and Short Duration Groups Using on Next-Generation Sequencing

Among 1,047 patients, 117 patients received initial chemotherapy with pemetrexed at Guangdong Provincial People's Hospital. Twenty-two plasma samples from 22 patients were collected to perform NGS. Their DOT ranged from 4.5 to 27.1 months. Thirteen patients were assigned to the long duration group (DOT > 6.9 months), whereas the short duration group (DOT ≤ 6.9 months) included nine patients. Their clinical characteristics are presented in Supplemental Table 2. The demographics and clinical characteristics of the patients were similar between the two groups in regard to age, sex, smoking status, and best response.

**Figure 5:**
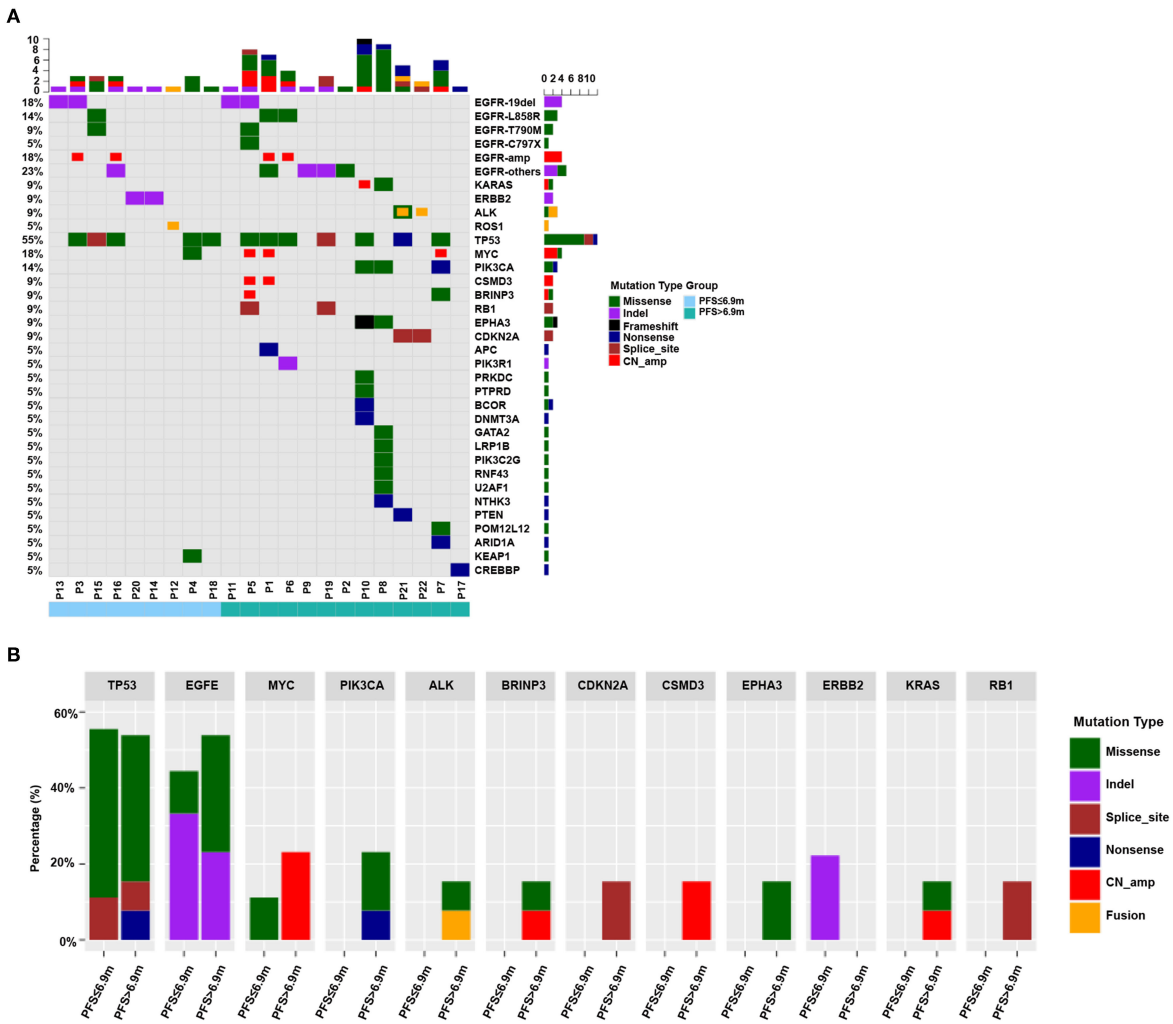
**(A)** Mutational frequency of 30 intersection genes analyzed in 22 patients by next-generation sequencing (NGS). The genes with high mutation rate included *TP53, EGFR, MYC*, and *PIK3CA*. **(B)** The differences of 12 genes mutational frequency between PFS > 6.9 months and PFS ≤ 6.9 months groups. *TP53, EGFR*, and *MYC* appeared recurrently in the two groups. *PIK3CA, ALK, BRINP3, CDKN2A, CSMD3, EPHA3, KRAS*, and *RB1* occurred only in the PFS > 6.9 months group whereas *ERBB2* mutation was detected only in the PFS ≤ 6.9 months group.

A total of 30 intersection genes were analyzed in these 22 patients, and the genes with high mutation rate included *TP53, EGFR, MYC*, and *PIK3CA*, accounting for 54.5% (12/22), 50.0% (11/22), 18.2% (4/22), and 13.6% (3/22), respectively (Figure 5A). We also compared the difference of 12 genes that were mutated in more than one patient between the two groups (Figure 5B). Three genes (*TP53, EGFR*, and *MYC*) appeared recurrently in the two groups. Genes that were mutated in the long duration group but not in the short duration group included *PIK3CA, ALK, BRINP3, CDKN2A, CSMD3, EPHA3, KRAS*, and *RB1*. *ERBB2* mutation was detected only in the short duration group.

## Discussion

Previous randomized controlled trials demonstrated that pemetrexed is effective and well-tolerated for patients with advanced non-squamous NSCLC (9–11). The PARAMOUNT trial was a relatively large randomized controlled trial that investigated whether continuation maintenance therapy with pemetrexed improved PFS after induction therapy with pemetrexed plus cisplatin in 539 patients randomly assigned to receive continuation maintenance therapy with pemetrexed plus best supportive care (*n* = 359) or with placebo plus best supportive care (*n* = 180) (9). The median PFS of pemetrexed during the induction plus maintenance period was 6.9 months, and the OS was 16.9 months. Therefore, that study provides high-level evidence for maintenance therapy with pemetrexed. Nevertheless, 94% of the patients enrolled in the PARAMOUNT study were Caucasian. Yang et al. reviewed the JMII and S110 studies to supplement the efficacy and safety data from PARAMOUNT on pemetrexed maintenance therapy in East Asian patients with non-squamous NSCLC (26). The median PFS during the entire period (induction plus maintenance) in the JMII and S110 studies was 5.7 months (95% CI, 4.4–7.3 months) and 6.83 months (95% CI, 5.78–7.98 months), which was consistent with that observed in the PARAMOUNT study (26). The median OS during the entire period was 20.2 months (95% CI, 16.7 to not available) in the JMII trial, whereas, in the S110 trial, the median OS could not be estimated due to a high censor rate (72.9%). Although the two studies suggested the efficacy of pemetrexed induction and maintenance therapy in East Asian patients, their samples were small. In our study, we collected a total of 1,047 patients from PAP throughout China, including 44 tertiary hospitals. It is the largest study performed so far to investigate the efficacy of pemetrexed initial chemotherapy in Chinese patients with advanced lung adenocarcinoma. The results showed that the median DOT of pemetrexed induction plus maintenance therapy was 9.1 months (95% CI: 8.5–9.8), and the median OS was 26.2 months (95% CI: 24.2–28.1), which were longer than that observed in clinical trials above. It added some evidence based on large samples showing that patients with lung adenocarcinoma can benefit from pemetrexed initial chemotherapy, especially for East Asians. This also provides a basis for which chemotherapy regimen may be the most appropriate combination with immunity inhibitors such as PD-1/PD-L1 inhibitor in the age of immunotherapy.

In the present study, the DOT was longer than the PFS observed in the PARAMOUNT, JMII, and S110 trials. One reason may be that our outcome DOT is different from PFS and is defined as the time from the start to the last treatment of pemetrexed. In clinical trials, the PFS is a common endpoint. Patients in clinical trials stop pemetrexed treatment once evaluated to have disease progression, according to RECIST. Nevertheless, in routine clinical practice, some patients still have a high likelihood of responding to maintenance therapy despite RECIST suggesting disease progression. Indeed, *EGFR* mutated patients with gradual and local progression after EGFR-tyrosine kinase inhibitors (TKIs) treatment failure still show persistent symptom benefit to continuing EGFR-TKIs treatment (27). In clinical practice, there are also some patients who receive pemetrexed treatment with gradual and local progression and showing persistent clinical benefit, although the disease was evaluated as progression according to RECIST. Although more details regarding the PFS and treatment beyond progression were not available, the DOT of 9.1 months in the present study was consistent with the PFS of 9.4 months reported by a real-world study from USA (12). Moreover, because of toxicity or other reasons, some patients may quit pemetrexed treatment when their tumors had not enlarged. Therefore, DOT may reflect the efficacy and safety of pemetrexed treatment more practically and objectively in clinical practice. The other reason may be that the present study was conducted in selected Chinese patients who might have a good prognosis and good response to pemetrexed since they completed the four-cycle induction therapy.

OS was also longer in the present study than that observed in the PARAMOUNT and JMII trials. Selected Chinese patients who received at least four cycles of pemetrexed treatment were enrolled in our study, which resulted in the longer OS besides the DOT. We also compared the clinical characteristics in the present study with those of the PARAMOUNT and JMII trials. Race and druggable target gene mutations were different between this study and the PARAMOUNT trial. Our study focused on Chinese patients, while 94% of patients were Caucasian in the PARAMOUNT trial, and we know that there are differences in genetic profile between the two races (28). *EGFR* mutations are more common in Asian populations than Caucasians (29). The rate of *EGFR* mutation in the present study is lower than that reported by Wu et al. (30) due to diverse gene detection methods with different sensitivity from 44 tertiary hospitals throughout China and certain percentages of patients with unknown *EGFR* status. Even so, *EGFR* mutated rate in our study was different from the PARAMOUNT trial. The multivariable analysis also showed that the *EGFR/ALK/ROS1* status and targeted therapy were independently associated with OS. This was similar to a previous study which reported that patients with an oncogenic driver mutation and who received a targeted therapy had survival benefits compared with those without oncogenic mutation or those who did not receive targeted therapy (31). The JMII trial was performed to evaluate the efficacy of pemetrexed and carboplatin, followed by pemetrexed maintenance therapy in chemotherapy-naïve patients with advanced non-squamous NSCLC in Japan. Out of 109 patients, 106 were evaluable for efficacy analysis. Although the median OS was 20.2 months (95% CI, 16.7 to not available), among 60 patients who received continuation maintenance with pemetrexed, the median OS from the beginning of induction treatment was not calculable. Besides genetic differences, more effective anticancer agents such as immunotherapy after pemetrexed treatment failure have been available from 2014 to 2018 when our study was conducted (32). The PARAMOUNT trial was carried out between 2008 and 2010, and the OS data cutoff date was in 2012 when antitumor treatment was more limited. The JMII trial was carried out between 2009 and 2010. All of these situations are potential reasons why the OS in our study was longer than that of previous clinical trials.

The multivariable analysis showed that the smoking status and ECOG PS were independently associated with DOT and OS, which was consistent with some previous studies (33–35). These factors might be potential clinical predictors for the effect of pemetrexed. Nevertheless, the differences of pemetrexed response among the patients could not be completely predicted by these clinical factors. This study tried to find potential genetic factors using NGS. A heterogeneous genetic profile was observed between the two groups by NGS. Mutations in *PIK3CA, ALK, BRINP3, CDKN2A, CSMD3, EPHA3, KRAS*, and *RB1* were only observed in the long duration group, whereas *ERBB2* mutation was only observed in the short duration group. But, these genes were not specific genetic profile benefit to pemetrexed treatment and may not predict the efficacy of pemetrexed. Further studies are needed to explore the predictor of the benefit to pemetrexed treatment in the future.

There are several limitations to our study. First, the present study excluded the patients who received pemetrexed for <4 cycles due to disease progression. This cannot represent the whole pemetrexed-treated patients in clinical practice in a real-world condition. Second, there are certain percentages of patients with unknown *EGFR, ALK, and ROS1* statuses, and the rate of patients with druggable target gene mutations received targeted therapy before pemetrexed treatment is low. Moreover, the exploration and comparison of gene profiles by NGS were conducted in a small sample of patients. All of these might make a bias to the conclusion. Third, more details regarding the PFS and treatment during beyond progression were not collected to prove the effectiveness of continuing pemetrexed chemotherapy beyond progression according to RECIST criteria. PFS and treatment beyond progression will have to be examined.

## Conclusion

This study shows that initial chemotherapy with pemetrexed is an effective regimen for advanced lung adenocarcinoma in selected Chinese patients. There is no specific genetic profile predicting the benefit of pemetrexed found by NGS. Biomarkers predicting the efficacy of pemetrexed need further exploration. More studies are needed to find a clinical treatment strategy of that chemotherapy combines with immunotherapy or targeted therapy.

## Data Availability Statement

The datasets presented in this study can be found in online repositories. The names of the repository/repositories and accession number(s) can be found at: The National Omics Data Encyclopedia (http://www.biosino.org/node/project/detail/OEP001054) and the European Genome-phenome Archive (https://www.ebi.ac.uk/ega/studies/EGAS00001004546).

## Ethics Statement

The studies involving human participants were reviewed and approved by the Research Ethics Committee of Guangdong Provincial People's Hospital, Guangdong Academy of Medical Sciences, School of Medicine, South China University of Technology. The patients/participants provided their written informed consent to participate in this study.

## Author Contributions

QZ and Y-LW conceived the idea and designed the experiments. L-HG and M-FZ analyzed the data together. L-HG wrote the manuscript. All authors, except QZ and Y-LW, were involved in the acquisition of data. All authors participated in the interpretation of the study results, drafting, critical revision, and approval of the final version of the manuscript.

## Conflict of Interest

The authors declare that the research was conducted in the absence of any commercial or financial relationships that could be construed as a potential conflict of interest.
