# Supplementary material for: Survival Benefit and Genetic Profile of Pemetrexed as Initial Chemotherapy in Selected Chinese Patients With Advanced Lung Adenocarcinoma
Source: Front Oncol. 2020 Sep 15;10:1568. doi: 10.3389/fonc.2020.01568 (PMC7522477; doi:10.3389/fonc.2020.01568)
Supplement: Supplementary file 1 [file Data_Sheet_1.docx]

**Supplemental Table 1.** The comparison of clinical characteristics and efficacy of pemetrexed between the present study and previous studies

| Variable | The present study (n=1047) | PARAMOUNT (n=359) | JMII (n=109) | S110 (n=27) |
| --- | --- | --- | --- | --- |
| Sex, n (%) |  |  |  |  |
| Male | 594 (56.7%) | 201 (56.0%) | 69 (63.3%) | 5 (18.5%) |
| Female | 453 (43.3%) | 158 (44.0%) | 40 (36.7%) | 22 (81.5%) |
| Age (years) |  |  |  |  |
| Median (range) | 59 (24-93) | 61 (32-79) | 63 (38-78) | 57 (29-75) |
| <65, n (%) | 758 (72.4%) | 238 (66.3%) | - | - |
| ≥65, n (%) | 289 (27.6%) | 121 (33.7%) | - | - |
| Ethnic origin, n (%) |  |  |  |  |
| Asian | 0 | 16 (4.5%) | 109 (100%) | 14 (51.9%) |
| African | 0 | 4 (1.1%) | 0 | 0 |
| White | 0 | 339 (94.4%) | 0 | 0 |
| Chinese | 1047 (100%) | 0 | 0 | 13 (48.1%) |
| Smoking status, n (%) |  |  |  |  |
| Smoker | 439 (41.9%) | 275 (76.6%) | 76 (69.7%) | 2 (7.4%) |
| Non-smoker | 608 (58.1%) | 82 (22.8%) | 33 (30.3%) | 25 (92.6%) |
| Unknown | 0 | 2 (0.6%) | 0 | 0 |
| ECOG PS, n (%) |  |  |  |  |
| 0-1 | 984 (94.0%) | 359 (100%) | 109 (100%) | 27 (100%) |
| 2 | 63 (6.0%) | 0 | 0 | 0 |
| Stage, n (%) |  |  |  |  |
| IIIB | 0 | 31 (8.6%) | 33 (30.3%) | 3 (11.1%) |
| IV | 1407 (100%) | 328 (91.4%) | 72 (66.1%) | 24 (88.9%) |
| Recurrence | 0 | 0 | 4 (3.6%) | 0 |
| Histology, n (%) |  |  |  | 0 |
| Adenocarcinoma | 1407 (100%) | 304 (84.7%) | 106 (97.2%) | 24 (88.9%) |
| Others | 0 | 55 (15.3%) | 3 (2.8%) | 3 (11.1%) |
| Gene status, n (%) |  |  |  |  |
| *EGFR* mutation |  |  |  |  |
| Positive | 266 (25.4%) | - | 24 (22.0%) | - |
| Negative | 568 (54.3%) | - | 63 (57.8%) | - |
| Unknown | 213 (20.3%) | 359 (100%) | 22 (20.2%) | - |
| *ALK* rearrangement |  |  |  |  |
| Positive | 59 (5.6%) | - | - | - |
| Negative | 580 (55.4%) | - | - | - |
| Unknown | 408 (39.0%) | 359 (100%) | - | - |
| Best tumor response |  |  |  |  |
| CR/PR | 477 (45.6%) | 166 (46.2%) | 38 (35.8%) | 10 (37.0%) |
| SD | 570 (54.4%) | 186 (51.8%) | 41 (38.7%) | 12 (44.4%) |
| PD | 0 | 1 (0.3%) | 20 (18.9%) | 3 (11.1%) |
| Unknown | 0 | 6 (1.7%) | 7 (6.6%) | 1 (3.7%) |
| Early death from toxicity | 0 | 0 | 0 | 1 (3.7%) |
| PFS (months), median (95%CI) | - | 6.9 (6.2-7.5) | 5.7 (4.4-7.3) | 6.8 (5.8-8.0) |
| DOT (months), median (95%CI) | 9.1 (8.5-9.8) | - | - | - |
| OS (months), median (95%CI) | 26.2 (24.2-28.1) | 16.9 (15.8-19.0) | 20.2 (16.7-NE) | - |

ECOG PS, Eastern Cooperative Oncology Group performance status; CR, complete response; PR, partial response; SD, stable disease; PD, progressive disease; PFS, progression-free survival; CI, confidence interval; DOT, duration of treatment; OS, overall survival; NE, not evaluable.

**Supplemental Table 2.** Clinical characteristics of 22 patients

| Characteristics | DOT≤6.9 months (n=9) | DOT>6.9 months (n=13) | *P* |
| --- | --- | --- | --- |
| Age (years), median (range) | 49 (33-79) | 55 (40-75) | 0.421 |
| Sex, n (%) |  |  | 0.099 |
| Female | 7 (77.8%) | 5 (38.5%) |  |
| Male | 2 (22.2%) | 8 (61.5%) |  |
| Smoking status, n (%) |  |  | 0.240 |
| Non-smoker | 9 (100%) | 10 (76.9%) |  |
| Smoker | 0 | 3 (23.1%) |  |
| ECOG PS, n (%) |  |  | - |
| 0 | 0 | 0 |  |
| 1 | 9 (100%) | 13 (100%) |  |
| Best response, n (%) |  |  | 0.203 |
| PR | 2 (22.2%) | 7 (53.8%) |  |
| SD | 7 (77.8%) | 6 (46.2%) |  |

DOT, duration of treatment; ECOG PS, Eastern Cooperative Oncology Group performance status; PR, partial response; SD, stable disease.
